# Electroacupuncture pretreatment mediates sympathetic nerves to alleviate myocardial ischemia–reperfusion injury via CRH neurons in the paraventricular nucleus of the hypothalamus

**DOI:** 10.1186/s13020-024-00916-y

**Published:** 2024-03-06

**Authors:** Jie Zhou, Bin Zhang, Xiang Zhou, Fan Zhang, Qi Shu, Yan Wu, Hui-Min Chang, Ling Hu, Rong-Lin Cai, Qing Yu

**Affiliations:** 1 College of Acupuncture and Moxibustion, Anhui University of Chinese Medicine, Hefei, 230012 China; 2 Center for Xin’an Medicine and Modernization of Traditional Chinese Medicine, Insitute of Health and Medicine, Hefei Comprehensive National Science Center, Hefei, 230601 China; 3 Institute of Acupuncture and Meridian Research, Anhui Academy of Chinese Medicine, Hefei, 230038 China; 4 Anhui Province Key Laboratory of Meridian Viscera Correlationship, Hefei, 230038 China; 5 Key Laboratory of Xin’an Medicine, Ministry of Education, Hefei, 230038 China

**Keywords:** Electroacupuncture pretreatment, Paraventricular nucleus of hypothalamus, CRH neurons, Myocardial ischemia- reperfusion injury, Neural mechanism

## Abstract

**Background:**

Myocardial ischemia–reperfusion can further exacerbate myocardial injury and increase the risk of death. Our previous research found that the paraventricular nucleus (PVN) of the hypothalamus plays a crucial role in the improvement of myocardial ischemia–reperfusion injury (MIRI) by electroacupuncture (EA) pretreatment, but its mechanism of action is still unclear. CRH neurons exhibit periodic concentrated expression in PVN, but further research is needed to determine whether they are involved in the improvement of MIRI by EA pretreatment. Meanwhile, numerous studies have shown that changes in sympathetic nervous system innervation and activity are associated with many heart diseases. This study aims to investigate whether EA pretreatment improves MIRI through sympathetic nervous system mediated by PVN^CRH^ neurons.

**Methods:**

Integrated use of fiber-optic recording, chemical genetics and other methods to detect relevant indicators: ECG signals were acquired through Powerlab standard II leads, and LabChart 8 calculated heart rate, ST-segment offset, and heart rate variability (HRV); Left ventricular ejection fraction (LVEF), left ventricular short-axis shortening (LVFS), left ventricular end-systolic internal diameter (LVIDs) and interventricular septal thickness (IVSs) were measured by echocardiography; Myocardial infarct area (IA) and area at risk (AAR) were calculated by Evans-TTC staining. Pathological changes in cardiomyocytes were observed by HE staining; Changes in PVN^CRH^ neuronal activity were recorded by fiber-optic photometry; Sympathetic nerve discharges were recorded for in vivo electrophysiology; NE and TH protein expression was assayed by Western blot.

**Results:**

Our data indicated that EA pretreatment can effectively alleviate MIRI. Meanwhile, we found that in the MIRI model, the number and activity of CRH neurons co labeled with c-Fos in the PVN area of the rat brain increased, and the frequency of sympathetic nerve discharge increased. EA pretreatment could reverse this change. In addition, the results of chemical genetics indicated that inhibiting PVN^CRH^ neurons has a similar protective effect on MIRI as EA pretreatment, and the activation of PVN^CRH^ neurons can counteract this protective effect.

**Conclusion:**

EA pretreatment can inhibit PVN^CRH^ neurons and improve MIRI by inhibiting sympathetic nerve, which offers fresh perspectives on the application of acupuncture in the management of cardiovascular disease.

**Supplementary Information:**

The online version contains supplementary material available at 10.1186/s13020-024-00916-y.

## Introduction

Myocardial ischaemia–reperfusion injury (MIRI) refers to further damage to the heart when blood flow is restored after a myocardial infarction. As of now, there is still no clinically effective way to treat this injury [1]. Acupuncture has been accepted as a representative traditional non-pharmacological treatment in an increasing number of countries. Acupuncture and moxibustion mainly stimulates the peripheral specific acupoints by needling the skin, causing the response of the central nervous system, thus regulating the function of internal organs. At the same time, human clinical studies have shown that acupuncture can have a significant impact on the cardiovascular system, providing effective treatment for a wide range of cardiovascular diseases [2–4]. The development of neurobrain science provides a good direction for thinking about explaining the mechanism of action of acupuncture in treating diseases [5].

The paraventricular nucleus (PVN) of the hypothalamus is a complex structure associated with neuroendocrine activity and autonomic function, and is critical for the regulation of neuroendocrine function and cardiovascular sympathetic nervous tension [6–10]. Meanwhile, autonomic nuclei in the brainstem and spinal cord can receive projections from neurons in the PVN’s small cell region, which activates the sympathetic nervous system, which controls heart rate [11]. Corticotropin-releasing hormone (CRH) neurons play an important role in neuroendocrine and stress responses. It is expressed in varied brain regions including the PVN, amygdala, arcuate nucleus, supraoptic nucleus, bluish pontine nucleus, and cerebral cortex, where the expression in PVN is more centralized [10]. About 70% of the adrenocorticotropic hormone (ACTH) response to acute stress can be inhibited by CRH antagonists or antibodies, indicating that CRH plays a major role in the hypothalamic-pituitary-adrenocortical (HPA) axis activation [12]. The sympathetic nervous system and the HPA axis become overactive when CRH is overexpressed in PVN neurons [13, 14]. A variety of heart diseases, including common ailments like hypertension, myocardial ischemia, and arrhythmias, as well as sudden infant death syndrome, have been linked to alterations in sympathetic innervation and activity [15, 16]. Arrhythmias in humans can result from sympathetic hyperinnervation or denervation [16]. Disruption of cardiac sympathetic innervation during mouse growth and development may be due to overexpression or deletion of the axon guidance signalling proteins SEMA3A and endothelin 1. And sympathetic disruption can lead to a variety of diseases, such as arrhythmia, heart failure, and even death [17–19].

Electroacupuncture (EA), as a method of acupuncture, has the advantages of definitive efficacy and less harm. Increasing evidence supports that EA is effective at relieving MIRI [20], however, the associated mechanism of action remains unclear. We have previously demonstrated that the PVN is a key mediator of the effect of EA pretreatment in alleviating MIRI [21]. The reports on PVN^CRH^ neurons as well as sympathetic nerves seem to suggest that both are definitely related to EA pretreatment to improve MIRI. So what is the connection between the three? Here, we sought to determine whether EA pretreatment alleviates MIRI by targeting CRH neurons in the hypothalamic PVN and whether this is achieved via sympathetic nerves.

## Materials and methods

### Animals

The experimental animals were clean-grade Sprague–Dawley (SD) male rats (8 weeks old, 200 – 250 *g*) purchased from Jinan Pengyue Experimental Animal Breeding Co., Ltd [Production License No.: SCXK (LU) 2019–0003] and raised in the Laboratory Animal Room of Key Laboratory of Xin’an Medicine Ministry of Education under controlled conditions (temperature: 24 ± 2 ℃, relative humidity: 50 – 60%, natural light 12 h alternating light and dark). The animals are free to eat and drink water. All experiments were conducted after 1 week of adaptive feeding. This study was performed according to the Guide for the Care and Use of Laboratory Animals of the Chinese National Institutes of Health. Approval for the study was granted by the Committee for the Care and Use of Research Animals of the Anhui University of Chinese Medicine (Approval No. AHUCM-rats-2022125).

### Model replication

Rats were fasted and deprived of water for 12 h before MIRI model preparation. Rats were positioned supine on an operating table and given 2% isoflurane (1 L/minute) to induce anaesthesia. The limbs and head were fixed and electrocardiography (ECG) was performed from standard limb leads II. The left anterior descending coronary artery, which is situated between the left atrium appendix and the pulmonary artery cone, was exposed after the skin was cut from the left third to fourth intercostal space, the muscular tissue was separated, the chest was opened, and the pericardium was separated. The left anterior descending coronary artery was ligated with a sterile No. 6–0 suture needle (depth of entry: 0.5 mm) 1 – 2 mm outside the starting point of the branch. After ligation, the ischemic myocardial wall became cyanotic and bulging, and the ST-segment of the standard limb lead II was bowed and elevated, suggestive of the presence of myocardial ischemia. After 30 min, the ligature was loosened, the chest cavity was closed, gases were dischared, and the ischemic coronary artery was reperfused for 120 min. When the ECG displayed ST-segment or T-wave elevation and a reduction in ST-segment elevation of greater than 50% following reperfusion, MIRI modelling was deemed successful. In the sham-operation group, the left anterior descending coronary artery was not ligated after the chest was opened, and only one puncture with a needle at the corresponding site was performed. Rats with an abnormal ECG before model replication, those that died during the experiment, and those for which modeling was unsuccessful were excluded from the study.

### EA pretreatment

The Shenmen (HT7) and Tongli (HT5) acupoints of the Hand Shao Yin heart meridian were selected for EA stimulation. Disposable sterile acupuncture needles (0.3 × 25 mm; Jiangsu Tianxie Acupuncture and Moxibustion Instrument Co., Ltd) were routinely sterilized before needling and then directly inserted 2–3 mm into the points. After needling, the “Shenmen” point was connected to the positive pole of the EA device (HANS-200A/100B; HANS, Beijing, China), and the “Tongli” point was connected to the negative pole,and were stimulated with a continuous wave of current at a frequency of 2 Hz and an intensity of 1.5 mA [22, 23]. The fake electroacupuncture (fEA) group chose non-acupuncture points in the tail of rats with the same electroacupuncture parameters [24]. The stimulation was repeated once a day for 30 min for 7 days. All EA operations were performed under isoflurane-induced anaesthesia, and the group that did not require EA was anaesthetised for the same duration using isoflurane.

### Animal grouping and experimental design

To clarify the mechanism of action associated with the impacts of EA pretreatment on MIRI, the experiment was divided into three parts.

### Experiment I

In the first part, 72 SD rats were randomly divided into four groups (Sham, Model, EA, and fEA) with 18 rats in each group. By observing the relevant indicators, it was determined which neurons were the key target of PVN in the process of EA pretreatment to alleviate MIRI.

### Experiment II

To further clarify whether CRH neurons are engaged in the anti-MIRI effect of EA pretreatment, we performed fiber-optic recording experiments on CRH neurons within the PVN. Eighteen SD rats were randomly divided into Sham, Model, and EA groups, with 6 rats per group. For virus injection, rAAV-CRH-CRE-WPRE-hGH-polyA and rAAV-EFla-DIO-GCaMP6s-EGFP-WPRE-pA were mixed in a 1:2 ratio and then injected unilaterally into the PVN (250 nL, 50 nL/min). In the Sham group, no model replication was carried out, and fiber-optic recordings were made directly 21 days after virus expression. In the Model group, modeling replication was carried out 21 days after virus expression and fiber-optic recordings were made immediately after the completion of modeling. In the EA group, EA pretreatment was performed on 14 day after virus expression for 7 consecutive days, and model replication was performed at the end of EA; fiber-optic recordings were made immediately after model replication. After the completion of the experiment, the rats in each group were perfused and the brains were removed to observe the virus injection and optical fiber embedding sites.

### Experiment III

To clarify the role of CRH neurons in the anti-MIRI effects mediated by EA pretreatment, 60 SD rats were randomly divided into five groups mCherry + Sham, mCherry + Model, mCherry + EA + Model, hM4Di + Model, and hM3Dq + EA + Model with 12 rats in each group. For virus injection, rAAV-CRH-CRE-WPRE-hGH-polyA and rAAV-EF1a-DIO-hM3Dq(hM4Di)-mCherry-WPREs were mixed in a 1:2 ratio and injected bilaterally (250 nL, 50 nL/min) into the PVN. In the hM4Di + Model group, the model was prepared on day 8 after intraperitoneal injection of clozapine N-oxide (CNO) for 7 consecutive days after 21 days of virus injection; in the hM3Dq + EA + Model group, CNO was injected intraperitoneally for 7 consecutive days after 21 days of virus injection, and EA was performed 40 min after the completion of the CNO injection, and the model was replicated on day 8 after the end of the EA; and in the remaining groups, an equal amount of CNO was injected. The anesthesia was induced by isoflurane, and the anesthesia time of each group was kept the same. At the end of the experiment, samples were taken to test the relevant indexes.

### Virus injection and fiber-optic burial

Virus injection: rats were fixed on a stereotactic device after isoflurane-induced anesthesia. An incision was made to expose the skull and holes were drilled in the target brain area based on the PVN coordinates ([relative to Bregma]; anteroposterior [AP]: − 1.5 mm, rostrolateral [RL]: ± 0.35 mm, dorsoventral [DV]: − 7.6 mm;), with the location of the nucleus pulposus, determined from the fontanel, serving as the base point. Virus (250 nL) was injected bilaterally at a rate of 50 nL/min and the needle was left in place for 10 min after completion of injection on both sides; after injection, seamed the incision with sterile suture. Postoperative continuous intraperitoneal injection of penicillin for 3 days to prevent infection. Fiber-optic burial: after unilateral injection of the virus the fiber was added via a fiber optic gripper and buried about 0.2 mm above the virus injection site. The entire surface of the skull was covered with instantaneous adhesive. Then covered with dental cement, ear tagged and documented experimentally, and ensure free access to water and feed.

### ECG recording

The electrocardiographic signals were acquired by PowerLab standard leads II. Then using LabChart 8 to analyse the ST-segment displacement values and the low-frequency (LF,0.04**–**0.15 Hz) /high-frequency (HF,0.15**–**0.4 Hz) ratios (LF/HF), respectively, for the first 5 min of the preparation of the MIRI model in the rats, for the 30 min of myocardial ischemia, and for the 120 min of reperfusion. Heart rate variability (HRV) at 120 min of reperfusion was analyzed using the Kubios HRV analysis software.

### Enzyme-linked immunosorbent assay

The standards were prepared, spiked and incubated in strict accordance with the instructions of the ELISA kits. The absorbance values were measured by the enzyme marker and the serum levels of norepinephrine (NE) and creatine kinase isoenzyme MB (CK-MB) in the samples of each group were calculated according to the generated standard curve.

### TTC-evans blue double staining

After the surgery was completed, the thoracic cavity of the rats was opened, and 1 mL of 2% Evans blue was injected into the apical part of the heart, which was allowed to perfuse the whole body with the blood, and the heart was removed after the limbs turned blue. Excess dye was washed off the hearts with pre-cooled saline, excess water was absorbed with filter paper, and the hearts were stored at – 20 ℃. After freezing, the heart muscle was cut into five 1-mm-thick slices at the ligation site. Sections were placed in TTC solution and incubated in an oven at 37 ℃ for 15 min, protected from light. Images were taken using a digital camera. The extent of myocardial infarction was calculated as the proportion of myocardial infarction area within the risk area, and the extent of risk was calculated as the proportion of the risk area within the total area of the cardiac section.

### Measurement of sympathetic electrical activity

Detection of sympathetic nerve discharges in rats using in vivo electrophysiologic techniques. The rat’s neck was depilated, the skin was incised along the anterior midline, and the muscle was separated layer by layer to locate the left carotid artery, which was separated from the trachea. A clear sympathetic ganglion can be seen. Hooked off the sympathetic nerve below the sympathetic carotid ganglia (SCG) and placed a cling film containing paraffin below it at a temperature of 36 ℃ to isolate surrounding tissues and maintain the activity of the sympathetic nerve. A glass separation needle was used to hook the nerve, and a platinum wire microelectrode was positioned below it at a 30° angle. The negative electrode was placed subcutaneously on the same side of the neck. The signal should be amplified using AM-300 preamplifiers (A-M Systems, Sequim, WA, USA), and it should be collected using LabChart 8.0 software (ADInstruments, China) and the PowerLab data acquisition system. Utilizing the LabChart 8.0 Spike Histogram Module, analyze the spike frequency (gain, 1000 Hz; low cut filter, 100 Hz; high-cut filter, 1000 Hz).

### Western blot

After 2 h of reperfusion, cardiac specimens were collected and tested for NE and Tyrosine hydroxylase (TH) protein expression. Samples were lysed in RIPA cell lysate (600 µL containing 0.6 mM PMSF) and then centrifuged at 12,000 rpm for 15 min to collect the supernatant. Gel configuration according to TRAKRA catalog. Add 5X SDS-PAGE protein uploading buffer at 1:4 to the collected protein samples. Heat in a boiling water bath for 15 min to fully denature the proteins. After the sample is cooled to room temperature, the protein sample can be directly sampled into the SDS-PAGE gel spiking wells. Add 5-10 uL per well. Electrophoresis at constant pressure 80 v for about 1 h. Pre-cut filter paper and PVDF membrane of the same size as the gel strip (pre-soaked in methanol for 2–3 min) were immersed in the membrane transfer buffer for 5 min. The membrane transfer device was placed in the order of anode plate, 3-layer filter paper, PVDF membrane, gel, 3-layer filter paper, and cathode plate from top to bottom, with the filter paper, gel, and PVDF membrane precisely aligned, and air bubbles removed at each step. Referring to the instructions of primary antibody, dilute with primary antibody diluent according to the appropriate ratio, and incubate overnight at 4 ℃ with slow shaking. Add the washing solution (PBST), wash for 10 min each time, wash three times in total. Referring to the properties of the primary antibody and the secondary antibody instructions, dilute horseradish peroxidase (HRP)-labeled secondary antibody with secondary antibody diluent according to 1:20,000. Incubate for 1.2 h at room temperature. Add washing solution (PBST) and wash for 10 min each time for a total of 3 times. Refer to the relevant instructions for the use of the ECL luminescent kit to detect proteins. The following antibodies were used: NE (Abcam, ab310335, 1:1000); TH (Abcam, ab315252, 1:3000); GAPDH (Zsbio, TA-08, 1:2000).

### Hematoxylin and eosin staining

Myocardial tissue to be tested was placed in paraformaldehyde fixative. Ethanol gradient dehydration: 75% ethanol 1.5 h, 5% ethanol 1.5 h, 95% ethanol 2.5 h, anhydrous ethanol I 0.5 h, anhydrous ethanol II 0.5 h. Xylene transparency: xylene I 15 min, xylene II 30 min. After paraffin embedding, the slices were cut into 5 μm-thick slices, dehydrated by ethanol gradient, and stained with hematoxylin–eosin in the routine procedure.

### Echocardiography

After 2 h of reperfusion, the wounds were sealed with absorbable sutures to avoid the influence of the sutures on the measurements. Rats were anaesthetised with inhaled isoflurane, chest hair was removed with depilatory agents, coupling agents were applied, and cardiac function was measured using a digital ultrasound device (Vinno 6 Lab, China) and an 18 MHz echocardiographic transducer. Left ventricular ejection fraction (LVEF), left ventricular short-axis shortening (LVFS), left ventricular end-systolic internal diameter (LVIDs), and interventricular septal (IVSs) thicknesses were then recorded using the M-mode.

### Immunofluorescence staining

Rat brain slices were immersed in PBS and washed three times, 5 min each wash. The brain slices were then blocked in a base solution containing 0.5% Tritonx-100 (Biosharp, Anhui, China) and 5% BSA (Spark Jade, Shandong, China) for 1.5 h. Subsequently, the slices were placed in 0.3% Tritonx-100 + 5% BSA base solution, the primary antibody was added dropwise at the ratio of 1:500, and the slices were incubated at 4 ℃ overnight. The next day, after washing three times with PBS, 5 min each wash, the slices were placed in a base solution containing 0.3% Tritonx-100 + 5% BSA, and the secondary antibody was added dropwise at a ratio of 1:500, followed by incubation for 2 h at room temperature, protected from light. After three PBS washes, the slices were sealed with an anti-fluorescence quenching solution containing DAPI. After staining was completed, three randomly selected image were photographed. The quantification of c-Fos-, CRH-, and GABA- positive neurons was performed in Image-Pro Plus 6.0. The following antibodies were used: rabbit anti-c-Fos (Beyotime, Cat. No. AF6489), rabbit anti-CRH (Proteintech, Cat. No. 10944-1-AP), rabbit anti-GABA (SIGMA, A2062-2ML), goat anti-rabbit IgG (Proteintech, Cat.No.SA00001-2), goat anti-rabbit IgG (Beyotime, Cat.No.A0516).

### Fiber-optic recording

After the surgery was completed, the rats were placed in special space containers. When they naturally awoke the fiber-optic connecting wire was attached to them and they were allowed to move freely. A multi-channel fiber-optic recording system (Chiaoxingo) was used to couple a 405 nm laser to a 470 nm blue light-emitting diode. Passage through the optical fiber was used to excite GCaMP6s fluorescence with a light intensity of 0.03 mW at the fiber tip. Band-pass filtered light was collected by a photomultiplier, and then an amplifier converted the current output from the photomultiplier to a voltage signal. Neuronal calcium signals were recorded in vivo using customized acquisition software at a frequency of 100 Hz. Further analysis was undertaken on MATLAB mat files to derive calcium signal (fluorescence) changes (ΔF/F), defined as (F − F0)/F0, which were presented as a graph of mean values or a heatmap.

### Statistical analysis

GraphPad Prism 8 was used to analyze all the data, and the results were presented as mean ± standard deviation (S.D.). One-way ANOVA with a 95% confidence interval was used to assess the differences between three or more experimental groups. Tukey’s multiple comparison post-hoc test was then performed. Paired or unpaired parameter t-tests were used to examine the differences between the two groups with a 95% confidence interval.

## Results

### EA pretreatment improved MIRI

**Fig.1:**
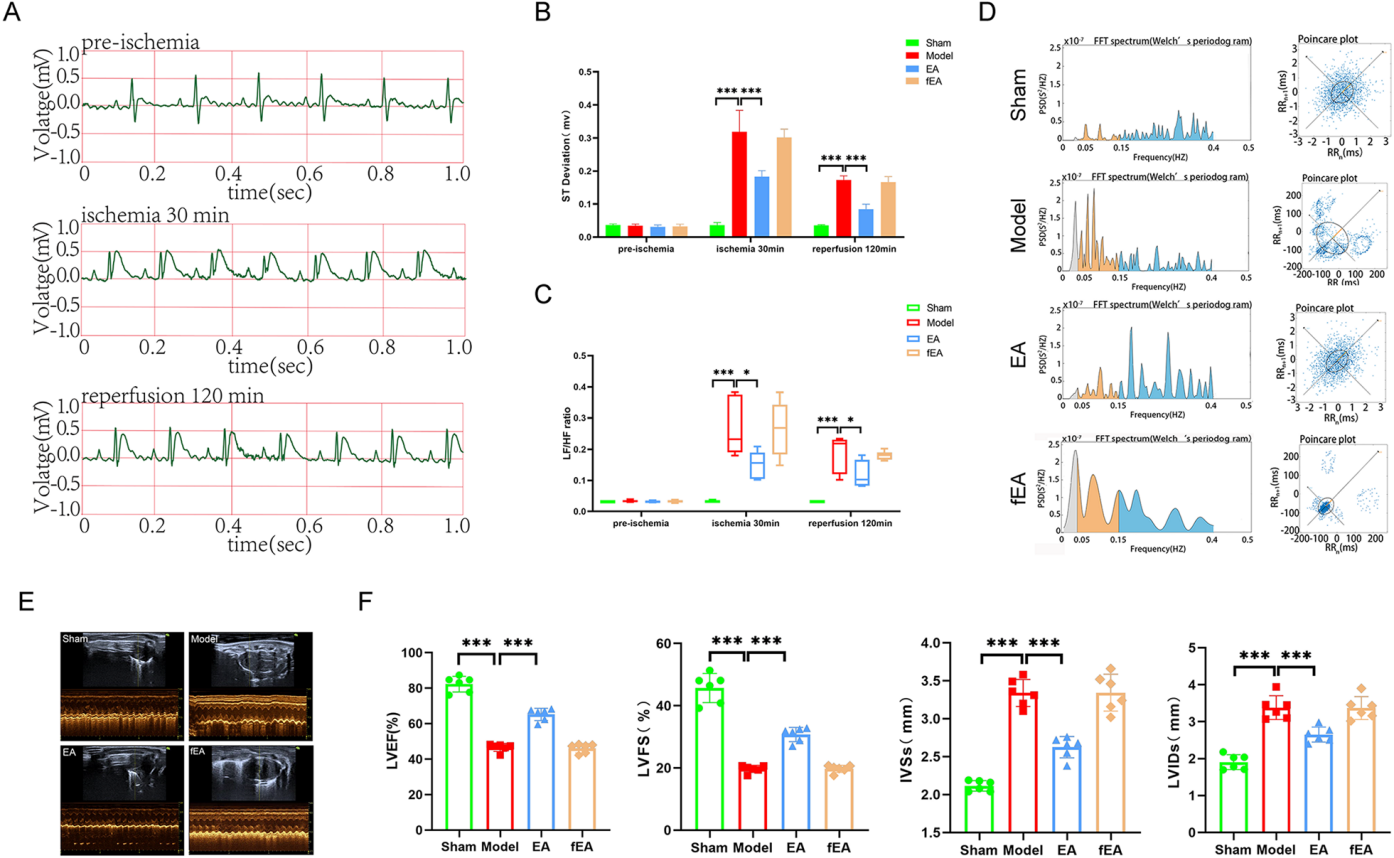
Effects of EA pretreatment on cardiac function in MIRI rats. **A** ECG recordings of the EA group. **B** The statistical analysis of ST deviations in each group of rats. Data are expressed as the mean ± SD. ***p < 0.001, n = 6 rats/group.** C** The statistical analysis of LF/HF ratio in each group of rats. Data are expressed as the mean ± SD. ***p < 0.001, *p < 0.05, n = 6 rats/group.** D** Comparison of frequency domain analysis in each group of rats. **E** Comparison of left ventricular echocardiography in each group of rats. **F** The statistical analysis of LVEF, LVFS, LVIDs, and IVSs values in each group of rats. Data are expressed as the mean ± SD. ***p < 0.001, n = 6 rats/group

The ECG results showed that the ST-segments of the rat ECG were lowest in the Sham group after successful model replication; the ST- segments of the rats in the EA group were lower than those of the Model group; however, the fEA group did not differ from the Model group, both 30 min postmyocardial ischemia and 120 min postreperfusion (Fig. 1A and Additional file 1: Fig S1). This suggested that EA pretreatment lowers the ST-segment and protects against MIRI at these two sampling time points, but fEA can’t (Fig. 1B). We also compared the HRV frequency domain of rats among the four groups, and found that rats in the Model group showed specific sympathetic excitatory waveforms, with a trend of increasing dispersion, indicative of increased sympathetic nerve excitability. However, these effects were suppressed in rats of the EA group, indicating that sympathetic nerve excitability, was decreased in this group compared with that in the Model group, while the fEA group remains unchanged (Fig. 1D). The LF/HF ratio showed a similar trend, namely, at 30 min postmyocardial ischemia and 120 min postreperfusion, the LF/HF ratio was increased in the Model group compared with that in the Sham group; the LF/HF ratio was decreased in the EA group compared with that in the Model group (Fig. 1C).

**Fig.2:**
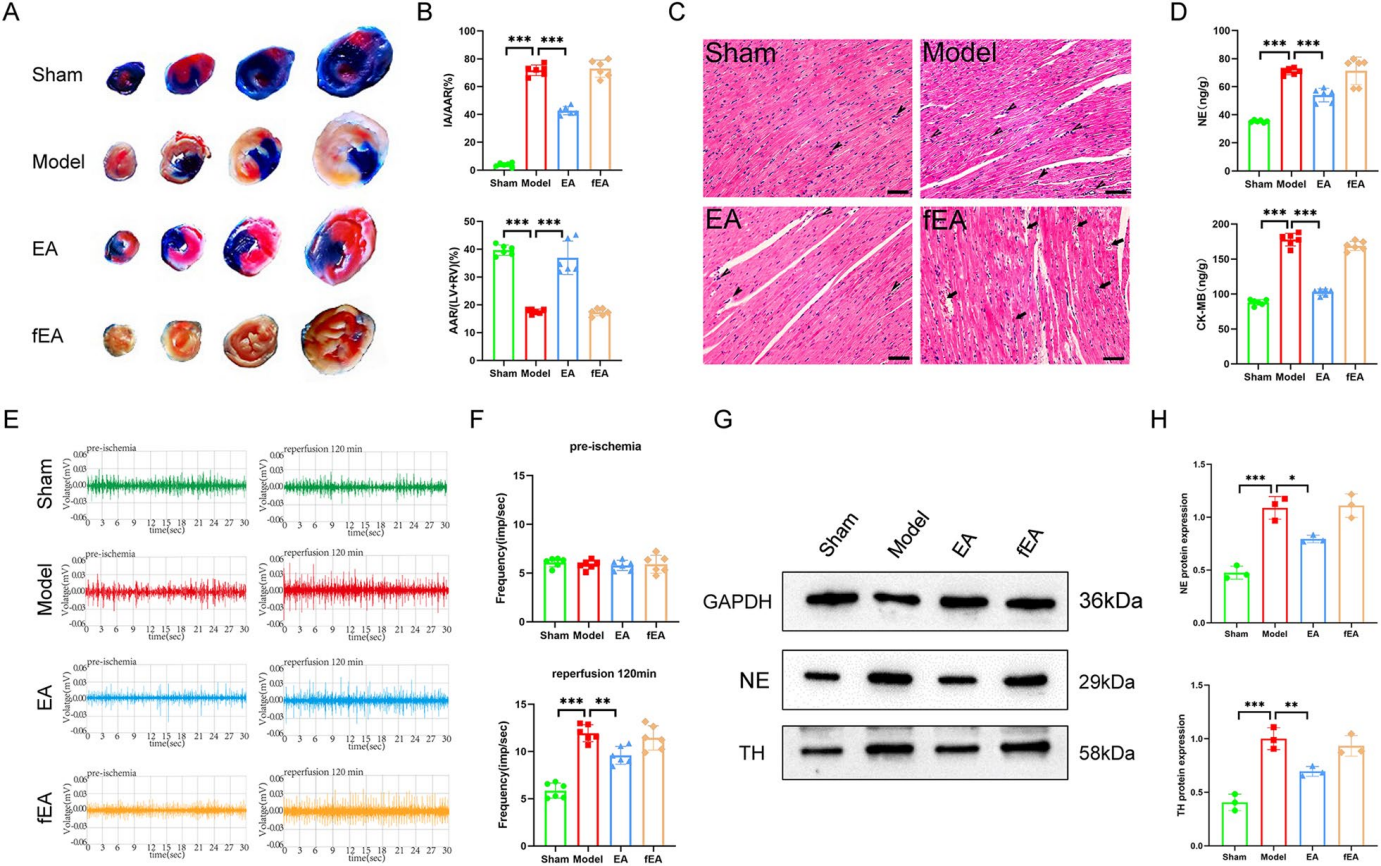
Effects of EA pretreatment on myocardial tissue and sympathetic nerves in MIRI rats.** A** Comparison of TTC-Evans blue double staining results of rat heart tissues in various groups. **B** The statistical analysis of area of myocardial infarction and area at risk in each group of rats. Data are expressed as the mean ± SD. ***p < 0.001, n = 6 rats/group. **C** Comparison of HE staining results of rat heart tissues in each group (magnification, × 20; scale bar, 100 µm). **D** The statistical analysis of NE and CK-MB values in myocardial tissue homogenates in each group of rats. Data are expressed as the mean ± SD. ***p < 0.001, n = 6 rats/group. **E** Comparison of original trajectory maps of sympathetic nerve activity in rats of various groups. **F** The statistical analysis of the frequency of sympathetic nerve discharges in each group of rats. Data are expressed as the mean ± SD. ***p < 0.001, **p < 0.01, n = 6 rats/group. **G** Comparison of NE and TH protein expression in each group of rats.** H** The statistical analysis of NE and TH protein expression in each group of rats. Data are expressed as the mean ± SD. ***p < 0.001, **p < 0.01, *p < 0.05, n = 3 rats/group

A single ECG result did not seem to fully justify the role of EA pretreatment. We then tested the relevant indicators that can reflect the function of the heart. We performed cardiac ultrasound on all four groups of rats (Fig. 1E). The results showed that, compared with rats in the Model group, those in the EA group had a higher LVEF, increased LVFS, smaller LVIDs and reduced IVSs thickness; similar results were observed between the Model and Sham groups. In contrast, there was no difference between the fEA group and the Model group (Fig. 1F). By TTC-Evans blue double staining, we found that myocardial infarct area (IA) was increased and area at risk (AAR) was decreased in the Model group of rats compared with the Sham group, in contrast to the EA group. The fEA group, on the other hand, maintained the same trend as the Model group (Fig. 2A, B). These findings suggested that EA pretreatment can lessen rat myocardial injury by increasing AAR, decreasing myocardial IA. In addition, HE staining results showed that the Sham group had normal myocardial tissue structure, orderly cardiomyocyte arrangement, and no cellular edema or myocardial fiber breaks could be observed. Meanwhile, the Model group and fEA group displayed uneven cardiomyocyte arrangement, severe cellular edema, myocardial fiber breaks, and extensive inflammatory cell infiltration. Compared with the Model group, the EA group displayed a more orderly cellular arrangement, reduced cellular edema, fewer myocardial fiber breaks, and less inflammatory cell infiltration (Fig. 2C). CK-MB contents can serve as a readout of myocardial injury, while NE responds to sympathetic excitability. Accordingly, we next measured the concentrations of CK-MB and NE in rat myocardium. We found that the levels of both factors were higher in the Model group than in the Sham group and were lower in the EA group than in the Model group. Same results as above, there was no difference between the fEA group and the Model group (Fig. 2D).

Lastly, we measured discharges in the sympathetic cardiac nerves following the inferior node of the SCG node to ascertain if EA pretreatment enhances MIRI through its effects on sympathetic nerves. (Fig. 2E). The findings demonstrated that prior to model replication, there was no variation in the frequency of nerve discharges among the four groups; after model replication, however, when contrasted with the EA group and the Sham group, the sympathetic nerve discharge frequency in the Model group was lower and higher, respectively. And fEA did not reduce the frequency of sympathetic nerve discharge (Fig. 2F). To further verify the involvement of sympathetic nerves, we examined the protein expression of NE and TH in the cardiac tissues of rats from all groups. NE and TH can respond directly or indirectly to sympathetic excitation [25, 26]. The results showed that the protein expression of NE and TH was increased in the cardiac tissues of rats in the Model group compared with the Sham group; there was no difference in the fEA group compared with the Model group, while the protein expression of NE and TH was decreased in the EA group (Fig. 2G, H). The above results suggested that EA pretreatment significantly attenuates MIRI, an effect that may be mediated via sympathetic nerves. However, fEA would not exert such an effect.

### EA pretreatment inhibited the activity of CRH neurons in the PVN

**Fig.3:**
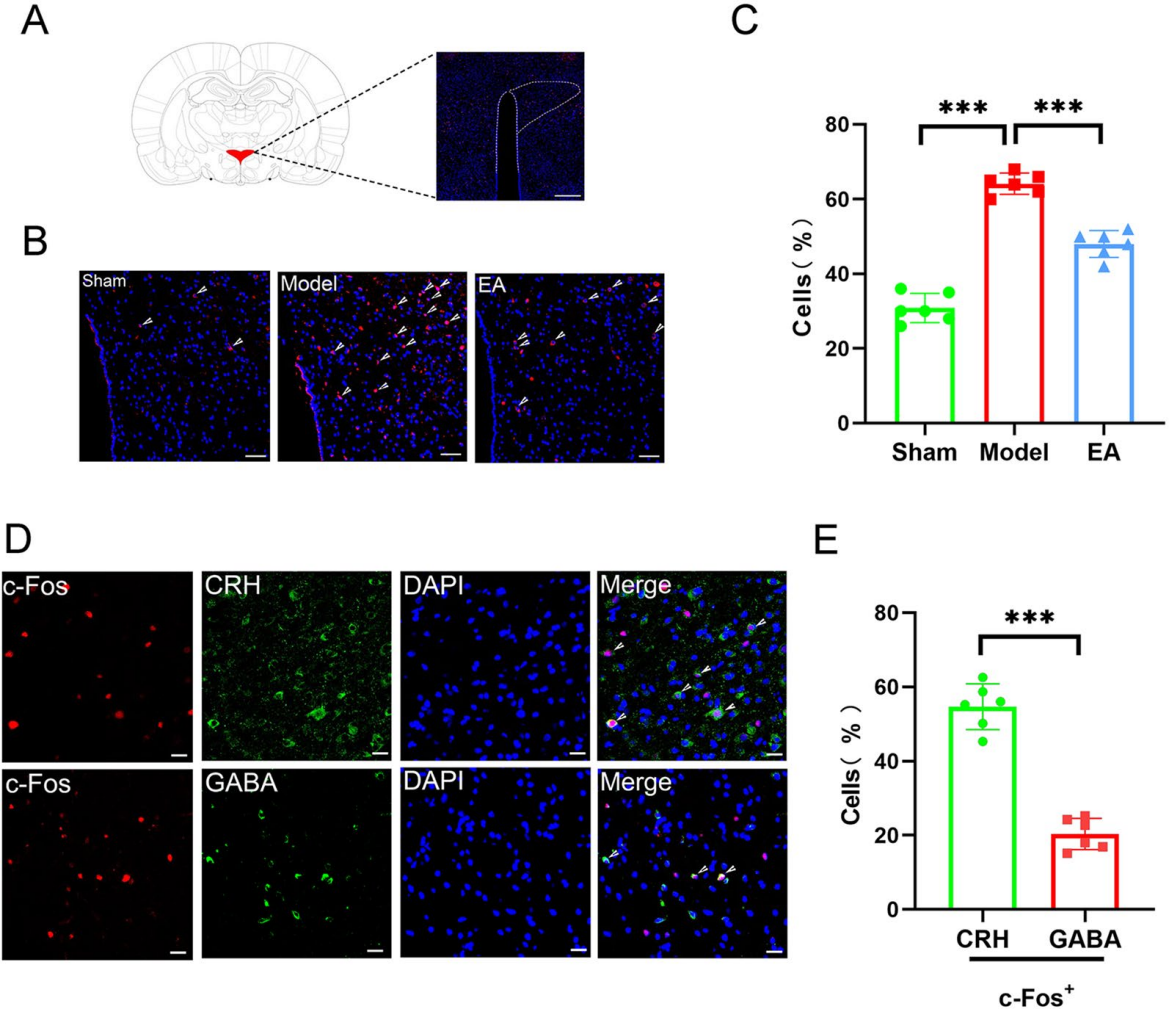
Results of immunofluorescence staining in the PVN. **A** Location of PVN (magnification, × 5; scale bar, 200 µm). **B** Comparison of c-Fos-positive neuronal expression in the PVN of rats in each group (magnification, × 20; c-Fos, red; DAPI, blue; scale bar, 50 µm). **C** The statistical analysis of the number of c-Fos + neurons in the PVN in each group of rats. Data are expressed as the mean ± SD. ***p < 0.001, n = 6 rats/group. **D** Comparison of CRH neurons and GABA neurons co-labeled with c-Fos in the PVN of MIRI rats (magnification, × 40; scale bar, 20 µm).** E** The statistical analysis of the number of CRH neurons and GABA neurons co-labeled with c-Fos in the PVN of MIRI rats. Data are expressed as the mean ± SD. ***p < 0.001, n = 6 rats/group

Research has shown that changes in different areas of the brain were caused by acupuncture at different points [27–29]. Analysis of c-Fos expression in the PVN region of rats in the three groups, demonstrated that c-Fos expression was upregulated in the Model group compared with that in the Sham group and reduced in the EA group compared with that in the Model group (Fig. 3A–C). These results suggested that EA pretreatment exerts its protective effects against MIRI by inhibiting c-Fos expression in the PVN brain region of rats. Next, we performed c-Fos CRH/GABA co-staining in the PVN. The results showed that the percentage of CRH-positive neurons co-labeled with c-Fos in the PVN was significantly higher than that of GABA-positive neurons co-labeled with c-Fos (Fig. 3D, E). This suggested that CRH neurons in the PVN may be a key target of EA pretreatment in its MIRI-attenuating effect. CRH/c-Fos double staining in the PVN of rats in the three groups further showed that the percentage of CRH-positive neurons that were co-labeled with c-Fos was significantly higher in the Model group than in the Sham and lower in the EA group than in the Model group (Additional file 1: Fig S2).

**Fig.4:**
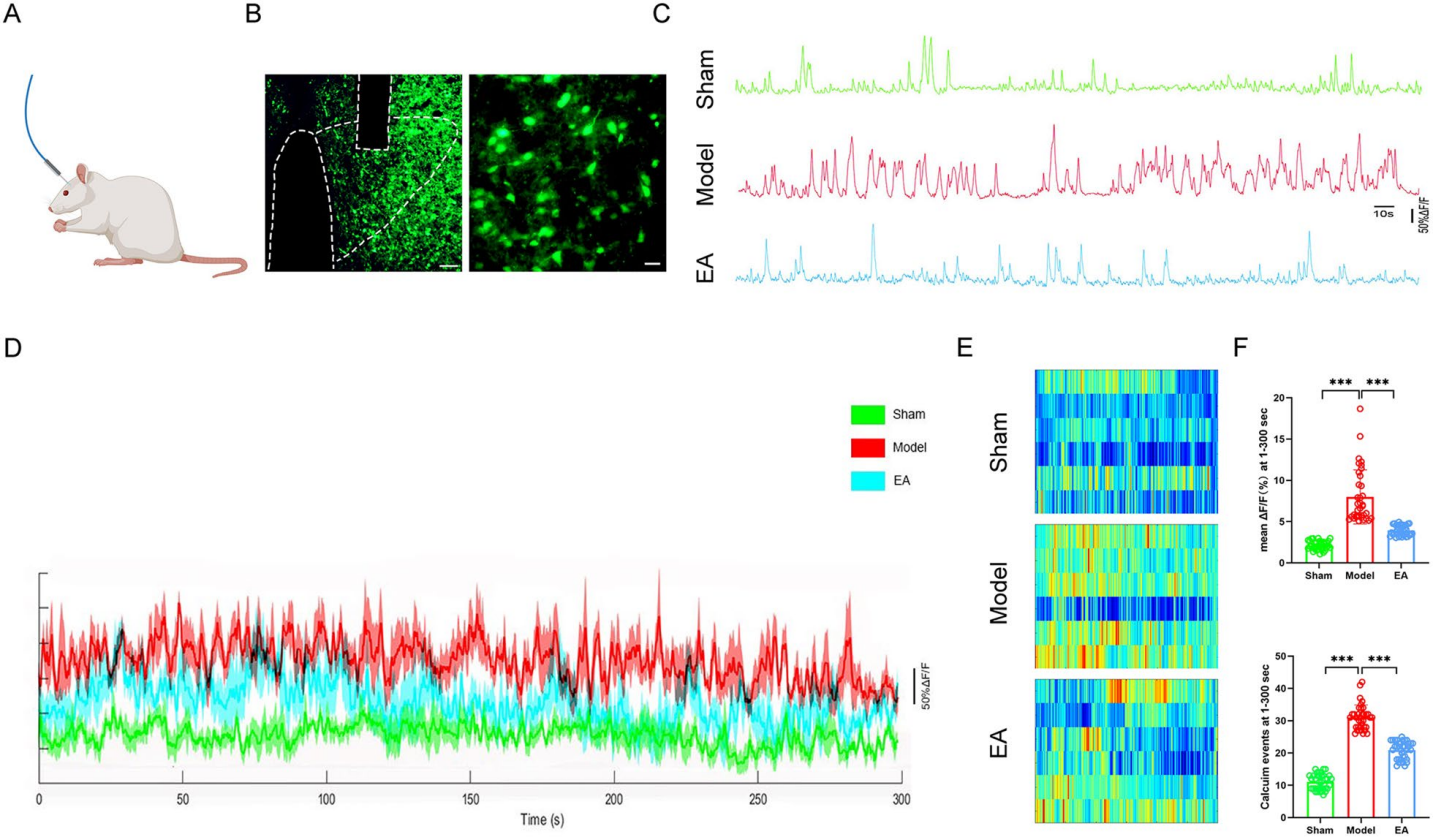
Results of fiber-optic recording of CRH neurons in the PVN. **A** Fiber optic burial diagram. **B** Fiber optic embedding sites and virus injection sites (magnification, × 10; scale bar, 100 µm; magnification, × 40; scale bar, 20 µm). **C** Comparison of the original trajectories of calcium activity in CRH neurons in 300 s in each group of rats. **D** Comparison of ΔF/F with time trajectory at 470 nm for each group of rats (n = 6).** E** Comparison of thermograms of ΔF/F over time trajectories in groups of rats (n = 6). **F** The statistical analysis of ΔF/F for 1–300 s and calcium events occurring in PVN^CRH^ neurons in each group of rats. Data are expressed as the mean ± SD. ***p < 0.001, n = 6 rats/group

We next performed optical fiber-based recordings in PVN^CRH^ neurons (Fig. 4A, B). The results indicated that the number of calcium transients and the transient amplitudes of signals in PVN^CRH^ neurons in the Model group were increased compared with those of the Sham group and decreased compared with those of the EA group (Fig. 4C, D). Analysis of a heatmap of ΔF/F over time showed that the calcium signaling energy of PVN^CRH^ neurons was higher in the Model group than in the Sham group, and was lower in the EA group than in the Model group (Fig. 4E). The fluorescence intensity in GCaMP6s + PVN^CRH^ neurons in the Model group was increased compared with that in the Sham group and decreased compared with that in the EA group. Quantification of calcium events per 300s showed that there were more calcium events in the Model group than in the Sham group, and a similar trend was detected compared with the EA group (Fig. 4F).

**Fig.5:**
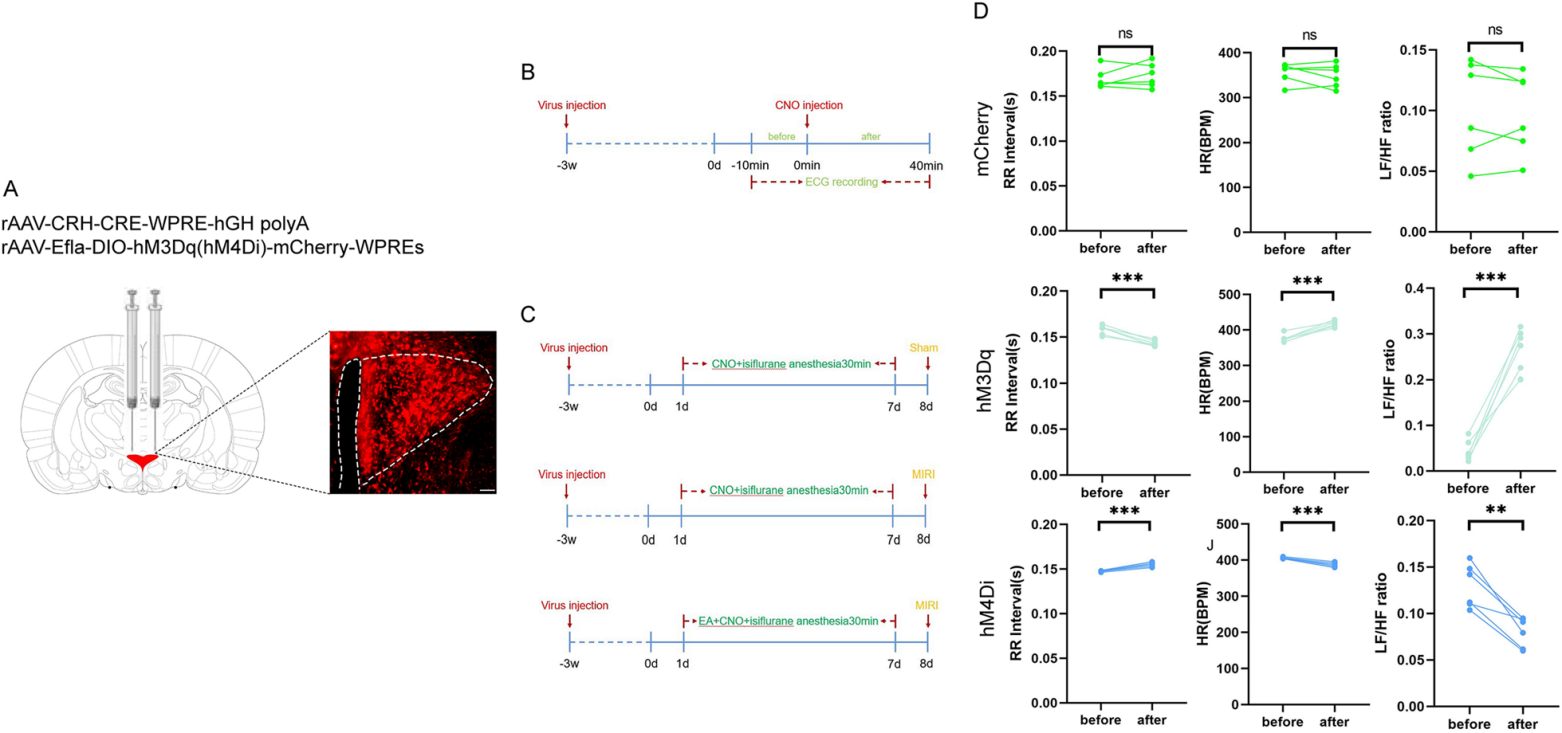
Results of chemogenetic regulation of PVN^CRH^ neurons. **A** Chemical genetic virus injection site (magnification, × 10; scale bar, 100 µm). **B** Experimental protocol for ECG recording under physiologic conditions in rats. **C** Experimental design diagram. **D** Comparison of various indices of ECG in rats injected with different kinds of viruses. Data are expressed as the mean ± SD. ***p < 0.001; **p < 0.01; ns, means no significance, n = 6 rats/group

**Fig.6:**
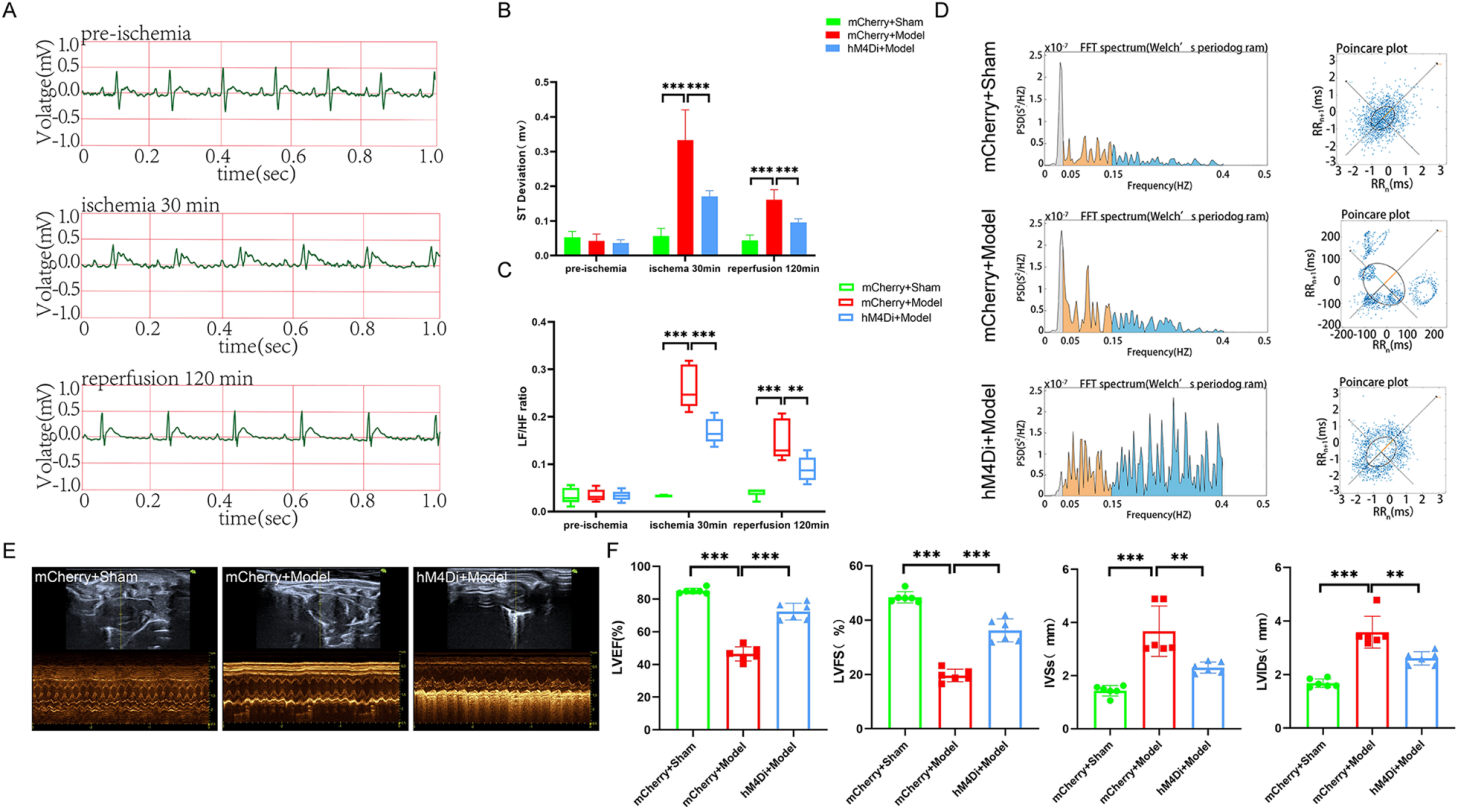
Effects of inhibition of PVN^CRH^ neurons on cardiac function in MIRI rats. **A** ECG recordings of the hM4Di + Model group. **B** The statistical analysis of ST deviations in each group of rats. Data are expressed as the mean ± SD. ***p < 0.001, n = 6 rats/group. **C** The statistical analysis of LF/HF ratio in each group of rats. Data are expressed as the mean ± SD. ***p < 0.001, **p < 0.01, n = 6 rats/group. **D** Comparison of frequency domain analysis in each group of rats. **E** Comparison of left ventricular echocardiography in each group of rats.** F** The statistical analysis of LVEF, LVFS, LVIDs, and IVSs values in each group of rats. Data are expressed as the mean ± SD. ***p < 0.001, **p < 0.01, n = 6 rats/group

The results of each of the above studies indicate that EA pretreatment has a direct inhibitory effect on CRH neurons in the PVN. These results indicate that EA pretreatment has a direct inhibitory effect on CRH neurons in the PVN. However, what role inhibiting CRH neurons in the PVN has on MIRI is not clear to us. To this end, we conducted the next step of the experiment. The inhibition of PVN^CRH^ neurons exerted similar effect to EA pretreatment in protecting against MIRI To further elucidate the mechanism of action of PVN^CRH^ neurons in the effects of EA pretreatment on MIRI, we used a chemical genetic approach to modulate these neurons. First, we designed a experiment to observe the relationship between PVN^CRH^ neurons and HRV under physiological conditions (Fig. 5A, B). We recorded ECGs in rats before and after injection of the chemogenetic virus with unloaded virus serving as a control. The results indicated that the activating PVN^CRH^ neurons can increase the heart rate and the LF/HF ratio and shorten the RR interval, whereas their inhibition exerted the opposite effects (Fig. 5D). This confirmed that PVN^CRH^ neurons regulate the heart by modulating HRV, which also reflects the existence of a link between PVN^CRH^ neurons and sympathetic nerves. Next, we performed different interventions on PVN^CRH^ neurons (Fig. 5C). According to the ECG data, the ST-segment was obviously lower in the hM4Di + Model group than in the mCherry + Model group and significantly higher in the mCherry + Model group as compared to the mCherry + Sham group. (Fig. 6A, B and Additional file 1: Fig S3). This suggested that, like EA pretreatment, the inhibition of PVN^CRH^ neurons plays a protective role against myocardial injury. Meanwhile, we also compared the frequency domains of HRV among the three groups of rats. The results showed that, compared with rats in the mCherry + Sham group, those in the mCherry + Model group exhibited specific sympathetic excitatory waveforms and the dispersion tended to show an increasing pattern, suggestive of enhanced sympathetic nerve excitability; however, these effects of modeling were mitigated in rats of the hM4Di + Model group indicative of decreased sympathetic nerve excitability (Fig. 6D). The LF/HF ratio showed a similar trend, that is, at 30 min postmyocardial ischemia and 120 min postreperfusion, the LF/HF ratio was increased in the mCherry + Model group compared with that in the mCherry + Sham group and decreased in the hM4Di + Model group compared with that in the mCherry + Model group (Fig. 6C).

**Fig.7:**
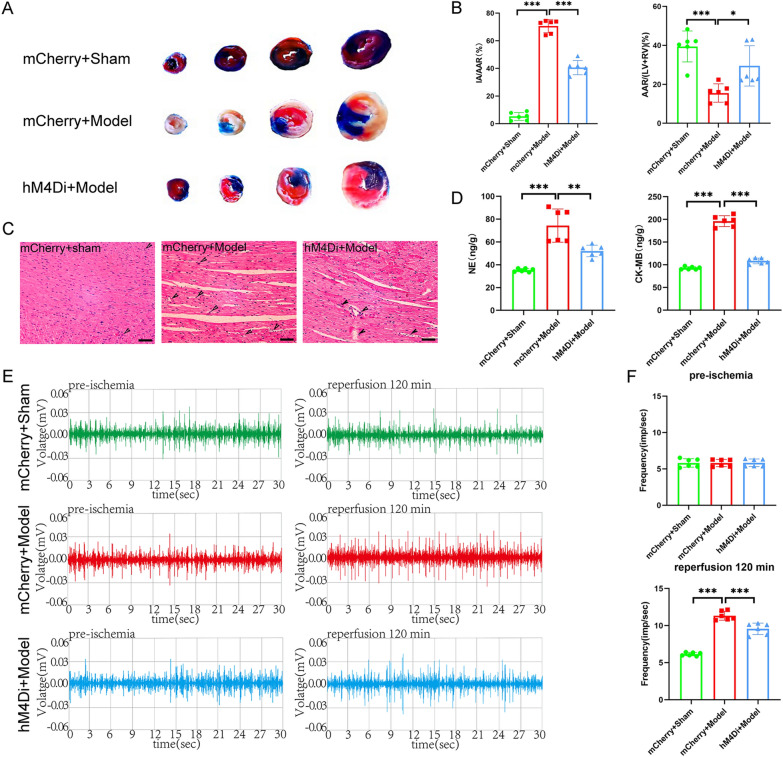
Effects of inhibition of PVN^CRH^ neurons on myocardial tissue and sympathetic nerves in MIRI rats. **A** Comparison of TTC-Evans blue double staining results of rat heart tissues in various groups.** B** The statistical analysis of area of myocardial infarction and area at risk in each group of rats. Data are expressed as the mean ± SD. ***p < 0.001, *p < 0.05, n = 6 rats/group. **C** Comparison of HE staining results of rat heart tissues in each group (magnification, × 20; scale bar, 100 µm). **D** The statistical analysis of NE and CK-MB values in myocardial tissue homogenates from various groups of rats. Data are expressed as the mean ± SD. ***p < 0.001, **p < 0.01, n = 6 rats/group. **E** Comparison of original trajectory maps of sympathetic nerve activity in rats of various groups. **F** The statistical analysis of the frequency of sympathetic nerve discharges in each group of rats. Data are expressed as the mean ± SD. ***p < 0.001, n = 6 rats/group

Inhibiting of PVN^CRH^ neurons in MIRI model rats not only increased the LVEF and LVFS, and reduced the LVIDs and IVSs thickness (Fig. 6E, F), but also reduced myocardial fiber breaks and intercellular edema (Fig. 7C). TTC-Evans blue double staining showed a significant increase in IA and a decrease in the AAR ratio in the mCherry + Model group compared with those of the mCherry + Sham group, effects that were reversed in the hM4Di + Model group (Fig. 7A, B). In addition, in the heart tissues of MIRI model rats, the inhibition of PVN^CRH^ neurons decreased the amounts of NE and CK-MB (Fig. 7D), and the frequency of sympathetic nerve discharges (Fig. 7E, F).

**Fig.8:**
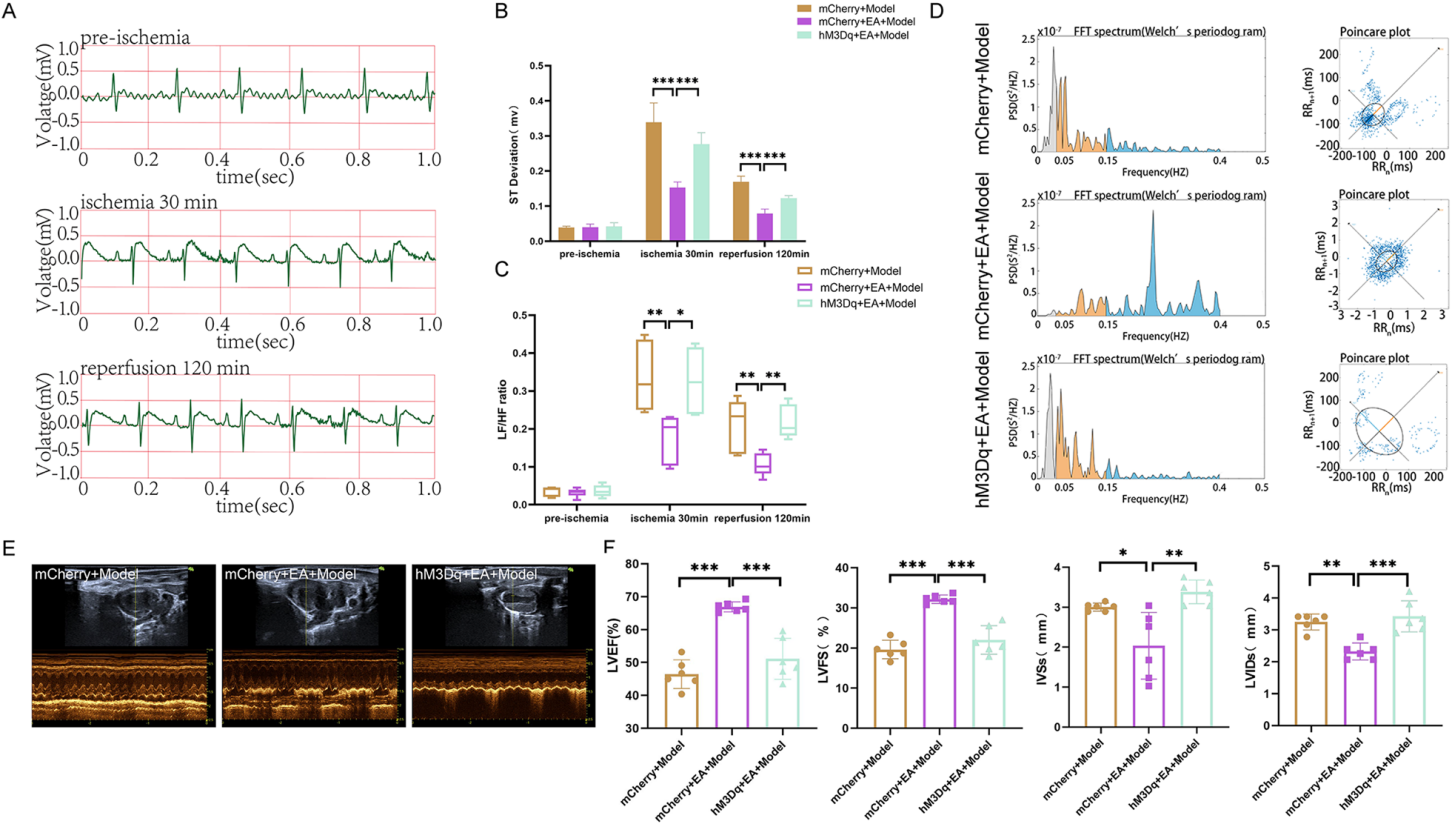
Effect of activation of PVN^CRH^ neurons on cardiac function in MIRI rats. **A** ECG recordings of the hM3Dq + EA + Model group. **B** The statistical analysis of ST deviations in each group of rats. Data are expressed as the mean ± SD. ***p < 0.001, n = 6 rats / group. **C** The statistical analysis of LF/HF ratio in each group of rats. Data are expressed as the mean ± SD. **p < 0.01, *p < 0.05, n = 6 rats/group. **D** Comparison of frequency domain analysis in each group of rats. **E** Comparison of left ventricular echocardiography in each group of rats. **F** The statistical analysis of LVEF, LVFS, LVIDs, and IVSs values in each group of rats. Data are expressed as the mean ± SD. ***p < 0.001, **p < 0.01, *p < 0.05, n = 6 rats/group

**Fig.9:**
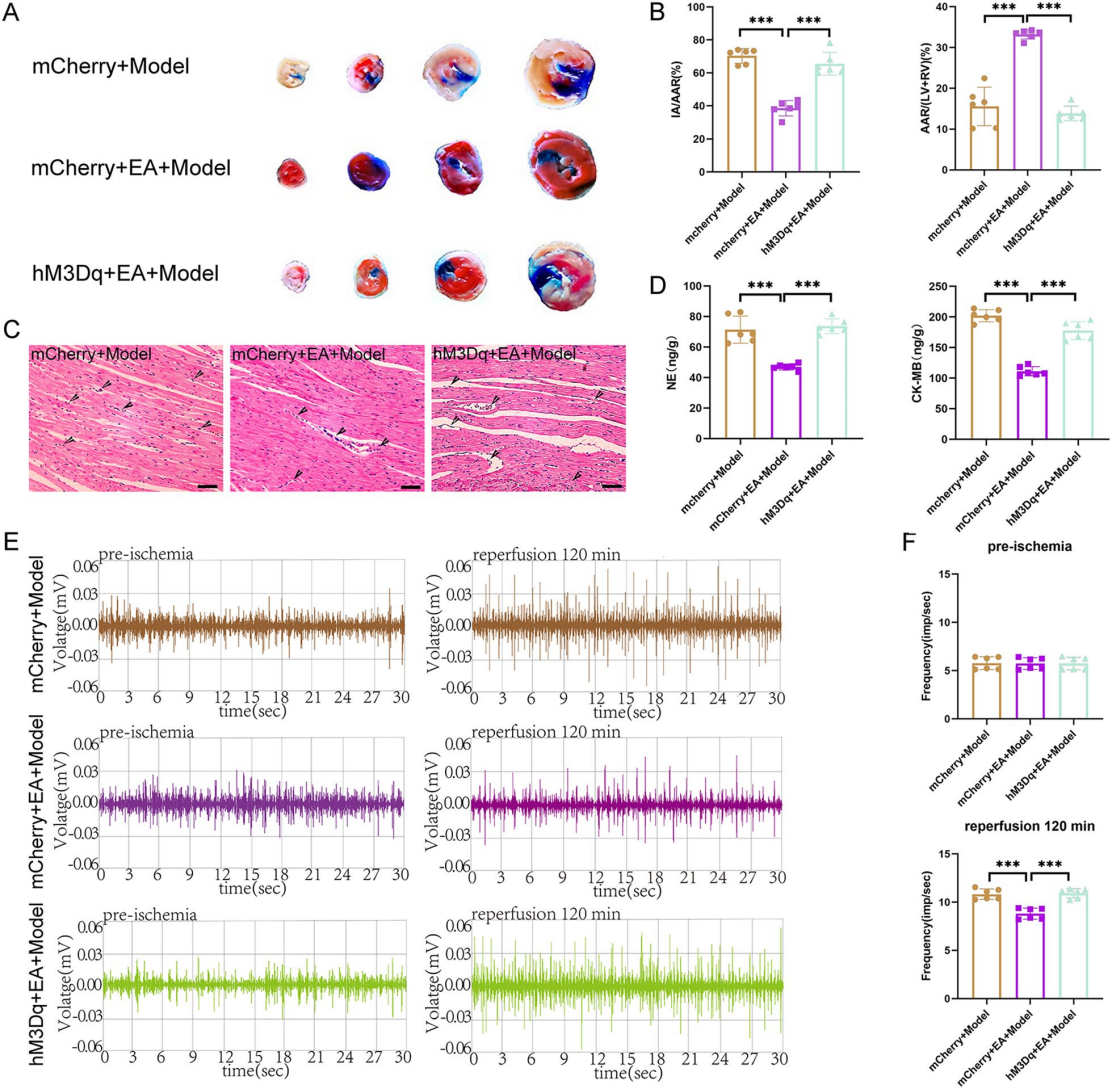
Effects of activation of PVN^CRH^ neurons on myocardial tissue and sympathetic nerves in MIRI rats. **A** Comparison of TTC-Evans blue double staining results of rat heart tissues in various groups. **B** The statistical analysis of area of myocardial infarction and area at risk in each group of rats. Data are expressed as the mean ± SD. ***p < 0.001, n = 6 rats/group. **C** Comparison of HE staining results of rat heart tissues in each group (magnification, × 20; scale bar, 100 µm). **D** The statistical analysis of NE and CK-MB values in myocardial tissue homogenates from various groups of rats. Data are expressed as the mean ± SD. ***p < 0.001, n = 6 rats/group. **E** Comparison of original trajectory maps of sympathetic nerve activity in rats of various groups. **F** The statistical analysis of the frequency of sympathetic nerve discharges in each group of rats. Data are expressed as the mean ± SD. ***p < 0.001, n = 6 rats/group

These findings implied that, like EA pretreatment, the inhibition of PVN^CRH^ neurons exerts a protective effect against MIRI in a sympathetic nerve-dependent manner. Despite this, the next step of the experiment was carried out for the sake of experimental rigour. The activation of PVN^CRH^ neurons attenuated the protective effect of EA pretreatment on MIRI To further validate our experimental results, PVN^CRH^ neurons were again chemically activated. The ECG results showed that the ST-segment was significantly lower in the mCherry + EA + Model group than in the mCherry + Model group or the hM3Dq + EA + Model group (Fig. 8A, B and Additional file 1: Fig S4). HRV frequency domain analysis showed that, compared with rats in the mCherry + Model group, those in the mCherry + EA + Model group exhibited suppression of sympathetic nerve excitatory waveforms with a decreasing dispersion trend, indicative of decreased sympathetic nerve excitability. Meanwhile, compared with mCherry + EA + Model rats, hM3Dq + EA + Model animals showed specific sympathetic excitatory waveforms, with an increasing dispersion trend, indicating that sympathetic excitability was enhanced (Fig. 8D). LF/HF statistics showed that at 30 min postmyocardial ischemia induction and 120 min postreperfusion, the LF/HF ratio was decreased in the mCherry + EA + Model group compared with that in both the mCherry + Model and the hM3Dq + EA + Model groups (Fig. 8C). Activating PVN^CRH^ neurons in MIRI model rats not only decreased the LVEF and LVFS and increased LVIDs and IVSs thickness (Fig. 8E, F), but also increased myocardial fiber breaks and intercellular edema (Fig. 9C). TTC-Evans blue double staining showed that there was a significant decrease in IA and a significant increase in the AAR in the hM3Dq + EA + Model group relative to that in the mCherry + Model group, effects that were reversed in the hM3Dq + EA + Model group (Fig. 9A, B). Additionally, The amounts of NE and CK-MB in the cardiac tissues of rats with MIRI models rose when PVN^CRH^ neurons were activated (Fig. 9D), as well the frequency of sympathetic nerve discharges (Fig. 9E, F).

The above results suggest that activating PVN^CRH^ neurons plays an opposite role to EA pretreatment. At this point, our experimental conjecture is reasonably and completely proven.

## Discussion

The pathogenesis of MIRI is complex and diverse and has not yet been systematically explained. However, it seems plausible that it may involve an interaction between the heart and the brain [30, 31]. Acupuncture is widely acknowledged to be an efficacious alternative complementary therapy with clear, long-lasting effects, and few contraindications [32]. Moreover, an increasing number of studies have investigated the therapeutic potential of acupuncture in the treatment of coronary heart disease [33, 34].

Meanwhile, the changes in brain activity induced by acupuncture provide a theoretical basis for exploring the neurobiological mechanisms underlying the effects of this technique in the treatment of cardiovascular diseases [35–37].

In our study, we first demonstrated that EA pretreatment significantly improved MIRI. We compared the ECG, HE staining of the heart, NE and CK-MB concentrations, and other related indexes among the three groups of rats and found that EA pretreatment had a good protective effect on cardiac function. Modern medicine proves that the organism itself has endogenous adaptive mechanisms. The purpose of pretreatment is to produce moderate stress in the body and activate the body’s endogenous adaptive mechanisms to prevent or mitigate disease-related injury [38]. The theoretical basis for the application of acupuncture pretreatment in the treatment of ischemic cardiovascular diseases is based on the premise that it promotes myocardial ischemia pre-adaptation. Acupuncture represents an effective means of inducing moderate, which can activate the body’s endogenous protective mechanisms and mobilize a variety of self-feedback and regulatory mechanisms to maintain internal homeostasis [39, 40]. Numerous studies have demonstrated that EA pretreatment has a protective effect on MIRI [41–43] and have highlighted its potential as an effective tool in the treatment of this condition [44]. This emphasizes the importance of, clarifying the mechanism of action relating to the ameliorative effects of EA pretreatment on MIRI.

The PVN of the hypothalamus functions as a nucleus for the integration of numerous autonomic and neuroendocrine inputs. It plays key roles in the regulation of the cardiovascular system [45, 46] as well as in the regulation of the autonomic nervous system (ANS) in various cardiovascular disease states [47, 48]. Acupuncture treatment involves the insertion of fine needles into the skin and underlying muscles and the manual or electrical stimulation of the needles. Thus, acupuncture stimulates somatic afferent nerves in the skin and muscles. Somatosensory information from the body is transmitted to cortical areas of the brain. Somatosensory fibers also project to various nuclei, including the brainstem of the hypothalamus, periaqueductal gray (PAG), and PVN. Acupuncture-stimulated somatosensory pathways activate these nuclei and regulate the imbalance between sympathetic and parasympathetic activity [49]. Acupuncture-stimulated somatosensory pathways activate these nuclei and modulate central structures and interconnected neural circuits in multiple brain regions, including the medulla oblongata, cerebral cortex, thalamus, and hypothalamus, such as the PVN-RVLM neural pathway. Activation or inhibition of these nuclei or neural circuits affects the imbalance between sympathetic and parasympathetic activity, which in turn affects cardiac function [50, 51]. Immunofluorescence double staining of the PVN in MIRI model rats revealed that a large number of CRH neurons were labeled with c-Fos proteins in this brain region, which suggested that these neurons might be central to the PVN-mediated regulation of cardiovascular function. Studies on CRH neurons have indicated that they play a pathogenic role in heart failure and hypertension [52, 53]; however, to the best of our knowledge, there is no report of their involvement in MIRI, it may be a new finding. CRH/c-Fos double staining of the PVN further showed the number of CRH-positive neurons that were co-labeled with c-Fos was significantly higher in the Model group than in the Sham group; however, this effect was significantly reversed by EA pretreatment further supporting that the ameliorative effects of EA pretreatment on MIRI may be mediated by CRH neurons. To further verify this possibility, we also performed fiber-optic recordings of PVN^CRH^ neurons in each group of rats, and found that calcium activity of in these neurons was significantly enhanced under the model; however, this effect was mitigated by EA pretreatment. This implied that PVN^CRH^ neurons indeed play an important role in the protective effects of EA pretreatment on MIRI. Interestingly, we also found that the frequency of sympathetic nerve discharges was increased in model rats, whereas EA pretreatment decreased this trend. This result was in line with the double staining results of the PVN which strongly supports that there is a connection between PVN^CRH^ neurons and sympathetic nerves.

It is well known that sympathetic nerves have a direct influence on cardiac function, and sympathetic hyperexcitation may further aggravate myocardial injury [54–56]. Our HRV analysis suggested that sympathetic nerve excitability was decreased in EA-pretreated animals relative to that in model animals. However, given that this parameter may not be a direct indicator of the excitability of sympathetic nerves [57], we performed a discharge test directly on the SCG. Meanwhile, it has been shown that some CRH neurons in the PVN project directly to the rostral ventrolateral medulla (RVLM) and the intermediolateral cell column (IML) of the gray matter of the spinal cord and participate in the regulation of the sympathetic nerve output by modulating the autonomic nuclei in these regions [58]. This observation reinforces our view that PVN^CRH^ neurons are linked to sympathetic nerves. To futher verify this conjecture, we undertook a SCG discharge assay in the chemical genetic modulation of PVN^CRH^ neurons. Our results showed that inhibition of PVN^CRH^ neurons resulted in a decrease in sympathetic nerve discharge frequency and a significant improvement in cardiac function in MIRI model rats, which phenocopied the effect of EA pretreatment; however, this protective effect of EA pretreatment was counteracted when PVN^CRH^ neurons were activated. These results strongly support our assumption that the ameliorative effects of EA pretreatment in MIRI are mediated by PVN^CRH^ neurons via sympathetic nerves.

In recent years, brain connectomics has been studied intensively and it has been gradually recognised that the connections between the body surface and viscera and between neurons in different brain regions are connections are effective means of clarifying the function exercised by brain network connections. This has also played an important role in explaining the neurological mechanisms underlying the specific presence of acupuncture. This also plays an important role in explaining the neurological mechanisms underlying the specific presence of acupuncture. In our study, we found that acupuncture signals altered the activity of PVN^CRH^ neurons, there may be targets of action for peripheral sensory-autonomic motor modulation in the PVN. Furthermore, our finding provides strong anatomical evidence for a central mechanism of cardiac autonomic regulation. Numerous studies have reported the existence of bidirectional communication between visceral organs and the brain [59, 60]. The generation of pinprick sensory signals is captured primarily by mechanically based injurious receptors. Mechanical injurious receptors are expressed in peripheral endings and induce peripheral sensory ganglia to generate neuroelectric signals that transmit the pinprick signal upwards to the brain. The brain integrates and processes and analyses the sensory signals, ultimately modulating visceral movement with autonomic output. This also argues nicely for our explanation of the central mechanism by which EA pretreatment relieves MIRI. However, there are many limitations to our study, and we have purely investigated CRH neurons in the PVN, the molecular mechanisms of which are not yet clear. Also, we have not clarified the mechanism of neural circuits related to PVN during EA pretreatment to alleviate MIRI. In our future research, we will make full use of modern neuroscience techniques such as instrumental viruses and optogenetics to provide new evidence for further clarifying the mechanism of the neural circuit in the regulation of cardiac function by acupuncture, and to lay the foundation for probing into the brain-regulatory mechanism of the acupuncture effect.

## Conclusions

In conclusion, in the present study, we demonstrated that EA pretreatment exerts an ameliorative effect on MIRI through the inhibition of PVN^CRH^ neurons and is achieved through the inhibition of sympathetic nerves. This explains the core mechanism involved in the ameliorative effects of EA pretreatment MIRI and lays the foundation for its use in clinical practice.

### Supplementary Information

**Additional file 1: Fig.S1** ECG recordings of rats in each group. A ECG recordings at various times in each group of rats. **Fig.S2** Results of CRH and c-Fos double staining. **A** Comparison of c-Fos co-labeling in CRH neurons in the PVN in each group of rats (magnification, × 10; scale bar, 100 µm). **B** The statistical analysis of the number of c-Fos co-labeling in CRH neurons in the PVN in each group of rats. Data are expressed as the mean ± SD. ***p < 0.001, n = 6 rats/group. **Fig.S3** ECG recordings of rats in groups with inhibition of PVN^CRH^ neurons. **A** ECG recordings at various times in each group of rats. **Fig.S4** ECG recordings of rats in groups with activation of PVN^CRH^ neurons. **A** ECG recordings at various times in each group of rats.

## Data Availability

The datasets used or analyzed throughout this study are available from the corresponding author upon reasonable request.

## Abbreviations

PVN: Paraventricular nucleus of hypothalamus
MIRI: Myocardial ischemia–reperfusion injury
EA: Electroacupuncture
fEA: Fake electroacupuncture
CRH: Corticotropin-releasing hormone
ACTH: Adrenocorticotropic hormone
HPA: Hypothalamic-pituitary-adrenocortical
ECG: Electrocardiography
CNO: Clozapine N-oxide
LF: Low-frequency
HF: High-frequency
HRV: Heart rate variability
NE: Norepinephrine
CK-MB: Creatine kinase isoenzyme MB
TH: Tyrosine hydroxylase
SCG: Sympathetic cervical ganglia
ANS: Autonomic nervous system
PAG: Periaqueductal gray
RVLM: Rostral ventrolateral medulla
IML: Intermediolateral cell column
