# Supplementary material for: Electroacupuncture pretreatment mediates sympathetic nerves to alleviate myocardial ischemia–reperfusion injury via CRH neurons in the paraventricular nucleus of the hypothalamus
Source: Chin Med. 2024 Mar 6;19:43. doi: 10.1186/s13020-024-00916-y (PMC10916233; doi:10.1186/s13020-024-00916-y)
Supplement: Supplementary file 1 — Additional file 1: Fig.S1 ECG recordings of rats in each group. A ECG recordings at various times in each group of rats. Fig.S2 Results of CRH and c-Fos double staining. A Comparison of c-Fos co-labeling in CRH neurons in the PVN in each group of rats (magnification, × 10; scale bar, 100 µm). B The statistical analysis of the number of c-Fos co-labeling in CRH neurons in the PVN in each group of rats. Data are expressed as the mean ± SD. ***p < 0.001, n = 6 rats/group. Fig.S3 ECG recordings of rats in groups with inhibition of PVNCRH neurons. A ECG recordings at various times in each group of rats. Fig.S4 ECG recordings of rats in groups with activation of PVNCRH neurons. A ECG recordings at various times in each group of rats. [file 13020_2024_916_MOESM1_ESM.docx]

**A key target for electroacupuncture pretreatment to alleviate myocardial ischemia-reperfusion injury via sympathetic nerves: CRH neurons in the paraventricular nucleus of the hypothalamus**

Jie Zhou^1,^ ^†^, Bin Zhang^1,^ ^†^, Xiang Zhou^1, †^, Fan Zhang^1^, Qi Shu^1^, Yan Wu^1^, Hui-Min Chang^1^, Ling Hu^3,5^, Rong-Lin Cai^2,3,4,5*,^, Qing Yu^3,4^[[1]](#footnote-1)^*^


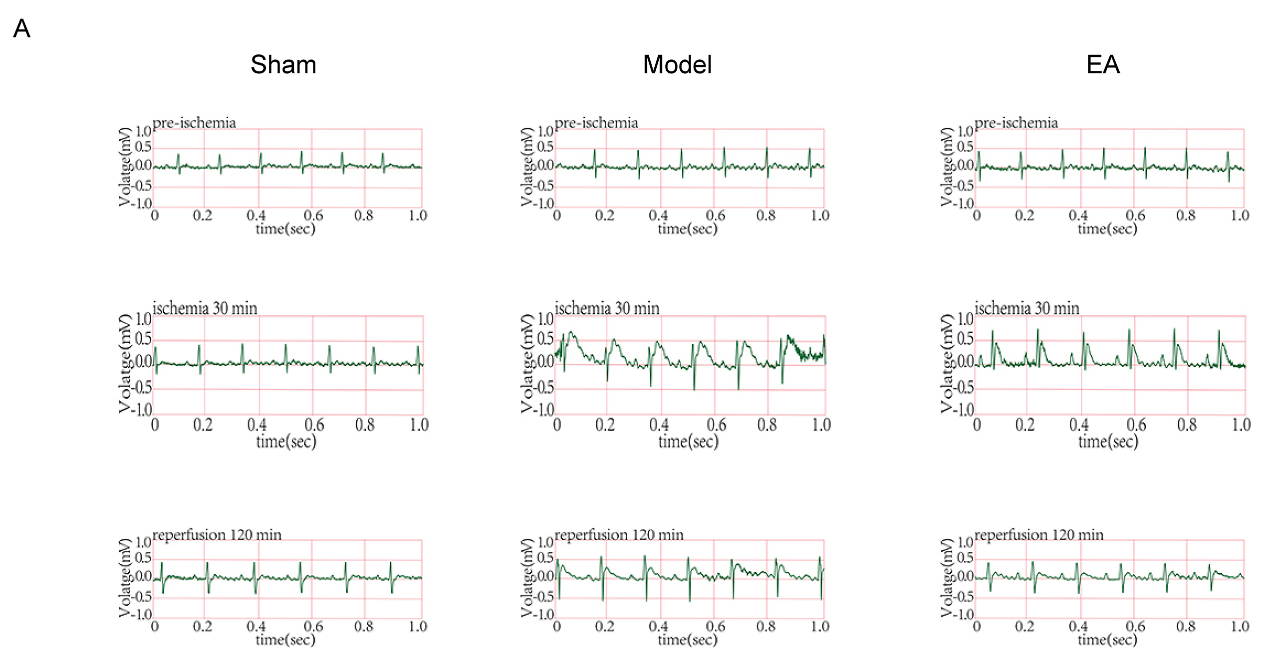


**Fig.S1** ECG recordings of rats in each group. **A** ECG recordings at various times in each group of rats.


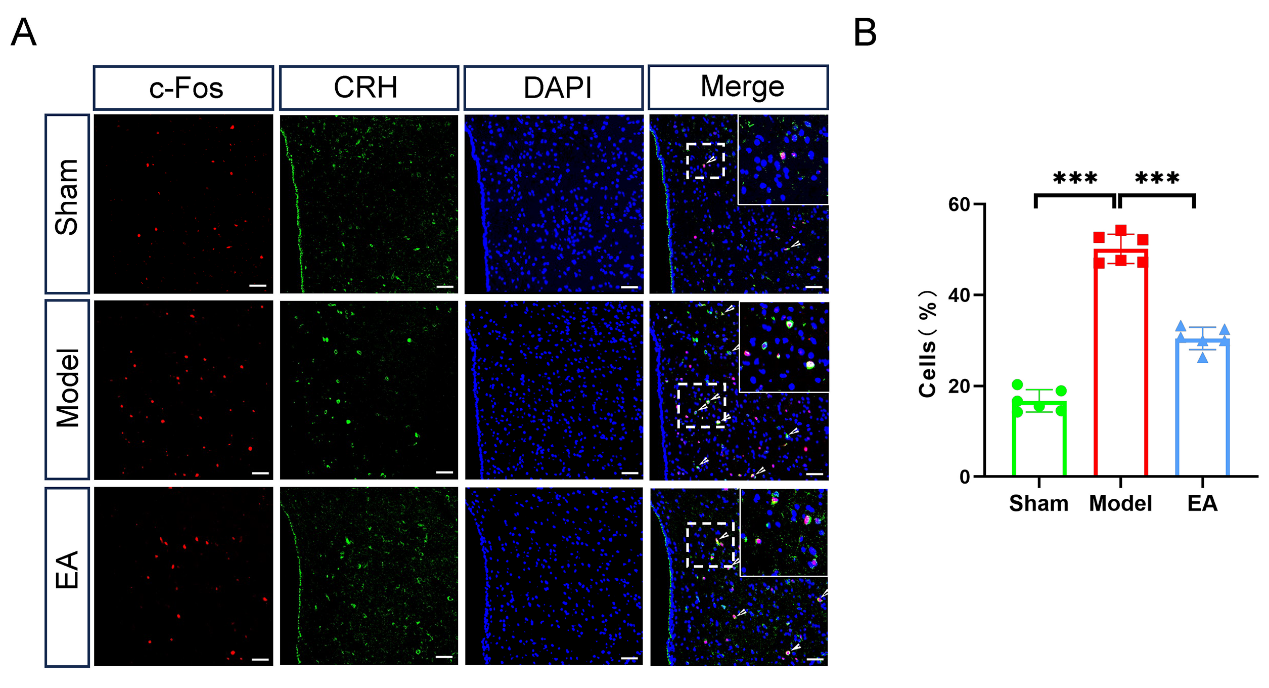


**Fig.S2** Results of CRH and c-Fos double staining. **A** Comparison of c-Fos co-labeling in CRH neurons in the PVN in each group of rats (magnification, × 10; scale bar, 100 µm). **B** The statistical analysis of the number of c-Fos co-labeling in CRH neurons in the PVN in each group of rats. Data are expressed as the mean ± SD. ****p*<0.001, n=6 rats / group.


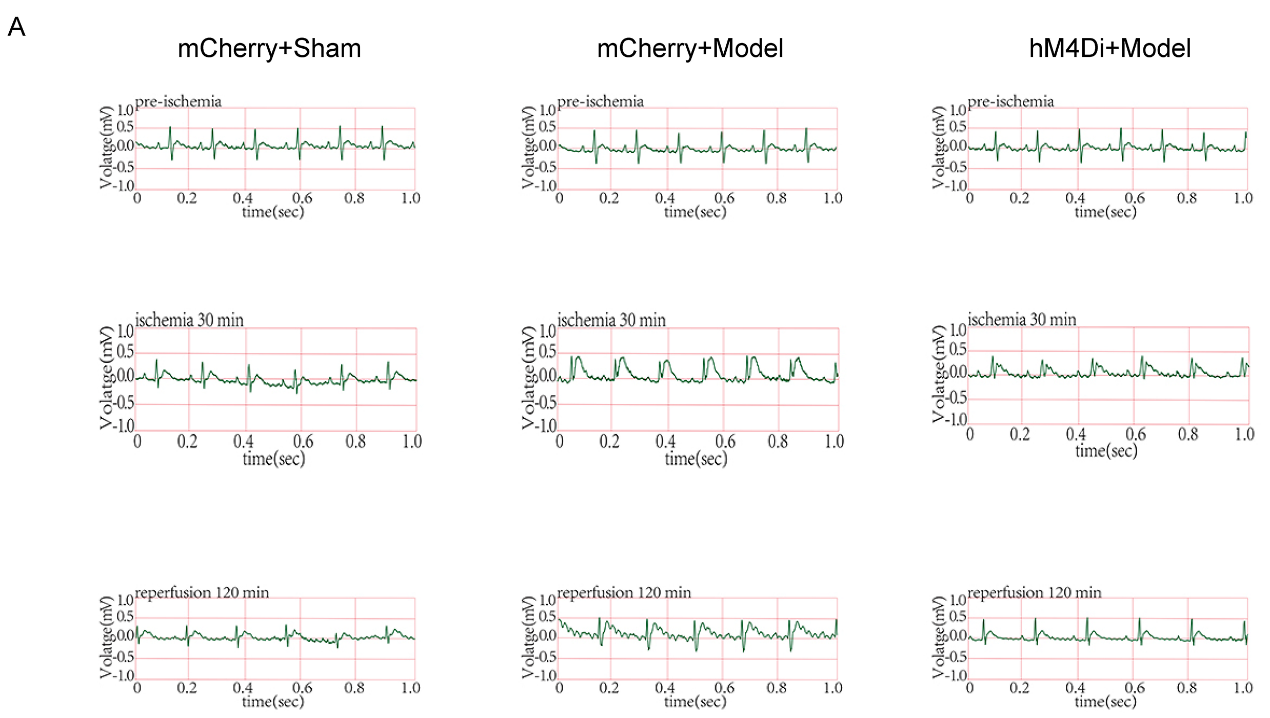


**Fig.S3** ECG recordings of rats in groups with inhibition of PVN^CRH^ neurons. **A** ECG recordings at various times in each group of rats.


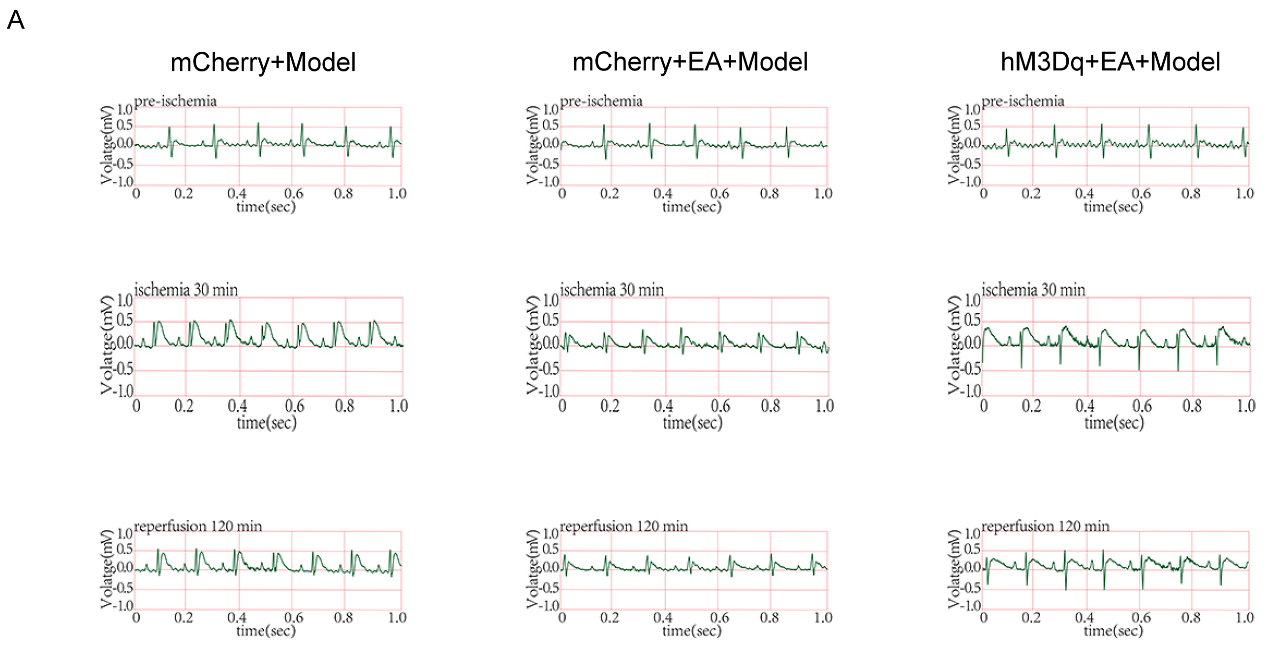


**Fig.S4** ECG recordings of rats in groups with activation of PVN^CRH^ neurons. **A** ECG recordings at various times in each group of rats.

1. †Jie Zhou, Bin Zhang and Xiang Zhou contributed equally to this study.

   *Correspondence:

   Rong-Lin Cai

   [ronglincai@ahtcm.edu.cn](mailto:ronglincai@ahtcm.edu.cn)

   Qing Yu

   [yuqing@ahtcm.edu.cn](mailto:yuqing@ahtcm.edu.cn%20)

   Full list of author information is available at the end of the article [↑](#footnote-ref-1)
